# Rapid Hemodynamic Deterioration and Death due to Acute Severe Refractory Septic Shock

**DOI:** 10.4021/jocmr2009.04.1238

**Published:** 2009-06-21

**Authors:** Abhijeet Dhoble, Won Chung

**Affiliations:** a Department of Internal Medicine, Michigan State University, East Lansing, Michigan, USA

## Abstract

Despite emergence of early goal directed therapy, septic shock still carries a high mortality. Gram negative septicemia is notorious for rapid deterioration due to endotoxin release. Multi-organ damage due to septic shock carries poor prognosis, and such patients should be managed aggressively with multidisciplinary approach. We present a fatal case of a patient with gram negative septicemia who rapidly deteriorated, and died due to acute refractory severe septic shock. This patient probably developed urosepsis secondary to severe urinary tract infection. He also had infiltrates on chest radiograph. He expired within fifteen hours of presenting to the emergency department. This case emphasizes the importance of early recognition and management of septic shock. Early goal directed therapy has shown to improve mortality. Broad spectrum antibiotics should be started within one hour depending on local immunity of organisms. This case also highlights the fact that despite optimized treatment, this entity has very high mortality rates.

**Keywords:**

Hemodynamic deterioration; Refractory septic shock; Gram negative septicemia

## Introduction

Sepsis is a clinical syndrome which is characterized by severe infection leading to systemic inflammation and widespread cellular injury [1]. Similar systemic response also occurs in the absence of an infection. This entity is different from culture negative sepsis syndrome, in which case there is an evidence or suspicion of infection, but blood or body fluid cultures are negative [2, 3]. In fact, blood cultures are negative in 30 to 80 percent of patients with sepsis depending on the severity of syndrome [3].

**Figure 1:**
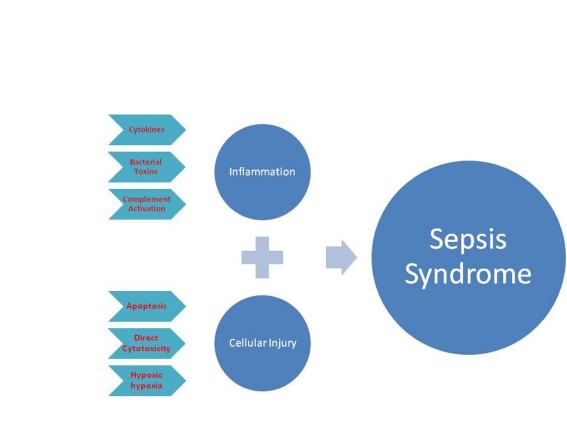
Pathogenesis of sepsis

Bloodstream infection is associated with high mortality rates. When bacteremia is associated with severe septic shock, gram negative and gram positive bacteria have comparable outcomes [4]. Dysregulation of anti and pro-inflammatory modulators is currently believed to be the center of pathogenesis of sepsis syndrome. Excessive spill of pro-inflammatory cytokines results in vasodilatation, increased endothelial permeability, leukocyte accumulation and neutrophils degranulation; leading to chain of events ensuing extensive tissue injury [5]. Cellular injury also plays an important role in the pathogenesis of sepsis through local hypoxia, apoptosis of injured cells, and direct cytotoxic effects [6]. Combination of excessive inflammation and cellular injury gives rise to full blown picture of sepsis syndrome (Fig. 1).

Gram negative septicemia is notorious for rapid deterioration due to endotoxin release [7]. Multi-organ damage due to septic shock carries poor prognosis, and such patients should be managed aggressively with multidisciplinary approach [8]. In spite of optimum treatment, refractory septic shock has very high mortality rates up to 50% [9]. Early goal directed therapy (EGDT) has shown to improve short and long term survival in these patients by 20%. EGDT is the mainstay of therapy while treating these patients [10]. We present a not very uncommon case of acute refractory gram negative septic shock, which rapidly deteriorated and died within 15 hours of his initial presentation to the emergency department.

## Case Report

A 65-years-old male patient presented to emergency department with complaints of progressive dyspnea, dysuria, and change in mental status in last six hours. The patient had a history of hypertension, type-2 diabetes mellitus, coronary artery disease, hypertriglyceridemia, peripheral vascular disease, chronic pancreatitis, gastroesophageal reflux disease, chronic back pain and depression. Surgical history included left popliteal-femoral bypass and coronary artery stenting. He had 25-pack-years history of smoking, but denied significant alcohol or illicit drug use. His customary medications were lantus insulin, glimeperide, verapamil, losartan, aspirin, rosuvastatin, fenofibrate, cilostazole, esomeprazole, dicyclomine, eszopiclone, venlafaxine, alprazolam, testosterone lotion, and hydrocodone-acetaminophen.

On physical examination, he was alert but not oriented, and in severe respiratory distress. Blood pressure (BP) was 160/94 mmHg in right arm, pulse rate was 140 per minute, respiratory rate was 30 breaths per minute, and temperature was 38.4^o^C. The Oxygen saturation via pulse oximeter was 86% while the patient was breathing 100% O_2_ by non-rebreather mask. There was decreased air movement at both lung bases, and consolidation at right lung base. Cardiac examination showed no abnormalities except tachycardia. The remainder of the examination was unremarkable.

**Figure 2:**
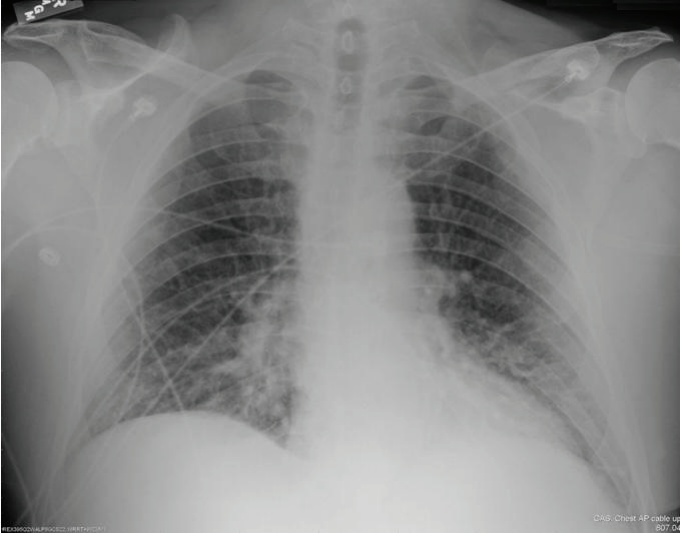
Chest radiograph at presentation

The initial labs and arterial blood gas (ABG) analysis are shown in Table 1 and 2. Serum lactate at the beginning was elevated at 4.1 mmol/L (normal: 0.5-1 mmol/L). Initial electrocardiogram showed sinus tachycardia with rate of 144 beats per minute. A chest radiograph showed increased interstitial markings and right lower lobe infiltrates (Fig. 2). Urine analysis study revealed presence of urinary tract infection and results are shown in Table 3. He was immediately started on fluid replacement, intravenous (IV) antibiotics which included levofloxacin, piperacillin/tazobactam and vancomycin, and high flow oxygen, and was transferred to medical intensive care unit (ICU) for further care.

**Table 1:** Initial laboratory values

Test	Value	Value range
Sodium	136 meq/l	135-145
Potassium	3.6 meq/l	3.5-4.9
Chloride	112 meq/l	96-110
CO_2_	11 mmol/l	20-30
Blood urea nitrogen	28 mg/dl	6.0-23.0
Creatnine	1.7 mg/dl	0.6-1.4
Total protein	6.4 g/dl	6.0-8.0
Albumin	3.7 g/dl	3.6-5.0
Magnesium	1.4 meq/l	1.3-2.2
Phosphorus	3.2 mg/dl	2.5-4.5
Calcium	8.3 mg/dl	8.0-10.5
White blood count	13,900/mm^3^	4-12
Hemoglobin	13.6 g/dl	12.6-16.5
Platelet count	119,000/mm^3^	150-400

**Table 2:** Arterial blood gas analysis

ABG	At presentation	After 12 hours	Normal Values
pH	7.26	6.9	7.35-7.45
PaO_2_ (mm Hg)	68	90	80-95
PaCO_2_ (mm Hg)	25	37	35-45
HCO_3_^-^ (mEq/L)	10.6	6	22-26
O_2_ Saturation	85%	84%	95-99%
FiO_2_	100%	100%	

**Table 3:** Urine analysis

Test	Findings	Normal values
White blood count	>100/hpf	0-2/hpf
Red blood count	1/hpf	0-2/hpf
Bacteria	many	none
Leukocyte esterase	Positive (large)	Negative
Nitrite	Positive (large)	Negative

Later on, patient went into severe respiratory distress, and endotrachial intubation was performed. Soon after intubation, patient went into pulseless electrical activity, and a code blue was called. Cardio-pulmonary resuscitation was performed as per standard protocol for 15 minutes, and patient came back to sinus rhythm. After the code, patient was hypotensive and required three vasopressors including epinephrine, norepinephrine and dopamine to maintain systolic BP more than 90. Oxygen saturation was 90% on 100% FiO_2_. After one hour, patient again went into cardiac asystole, then ventricular fibrillation, and then pulseless electrical activity. Successful resuscitation was done for 15 minutes. After this second code, the systolic BP was in 80s, and IV phenylephrine was added. Hear rate was 146 beats per minute, and oxygen saturation 88% on 100% FiO_2_. The laboratory results showed lactate level of 12.1 mmol/L; transaminase in 1000's and blood urea nitrogen and creatinine of 53 mg/dl and 4.9 mg/dl respectively. Serum troponin level was 2.92 ng/ml (normal ≤ 0.04 ng/ml). Arterial blood gas (ABG) revealed worsening metabolic acidosis.

An emergent two-dimensional echocardiogram was obtained and revealed normal left and right ventricular function. His left ventricular ejection fraction was found to be 50%, and there was no pericardial effusion, or right ventricular strain. Patient was started on IV hydrocortisone, and anidulafungin was added to cover for fungal pathogens. A decision was also made to start patient on activated protein C infusion considering high risk of death. In addition, bicarbonate drip was also started.

Twelve hours after his admission to this hospital, patient started showing signs of anoxic brain injury. At that time, he was on maximum doses of five vasopressors, but was still hypotensive and tachycardic. The oxygen saturation decreased to 88% on 100% of FiO_2_. ABG at that time is shown in Table 2. Family meeting was called, and they requested for withdrawal of care. Thirty minutes later, care was withdrawn and patient expired within 10 minutes of extubation and stopping IV medications. Family refused autopsy. The next day, two out of four blood cultures were positive for Escherichia coli.

## Discussion

There are approximately 750,000 cases of sepsis diagnosed in the United States annually [11]. It occurs in approximately two percent of hospitalized patients and up to 75 percent of intensive care unit patients [12-15]. Hospital mortality rate for sepsis patients ranges from 20 to 50 percent [12]. Despite an 8 percent per year increase in the incidence of sepsis, mortality rate have declined over the past 20 years [12-14]. Among patients with sepsis, the severity of disease appears to be increasing. In one large retrospective analysis, the proportion of patients with severe sepsis increased from 26 to 44 percent over a decade [13, 14]. Despite this, hospital case-fatality rates declined dramatically [15]. The trends of increasing incidence and improved survival have also been observed for septic shock [12-15]. Mortality rates increase stepwise according to disease severity [12-14].

The contribution of various infectious organisms to the burden of disease has changed over time. Gram negative bacilli were the predominant organisms associated with nosocomial bacteremia in the US prior to the 1980s [16]. Gram positive aerobes now outnumber Gram negative organisms [11, 17], especially the emergence of *methicillin resistant staphylococcus aureus*. Although the percentage of gram negative bacillary bacteremia has decreased, these cause serious multidrug resistance problems increasing mortality rate [18], and is usually associated with at least one comorbid condition [19]. In 2003, gram-negative bacilli were responsible for 24 percent of nosocomial bacteremias in ICUs [16].

**Figure 3:**
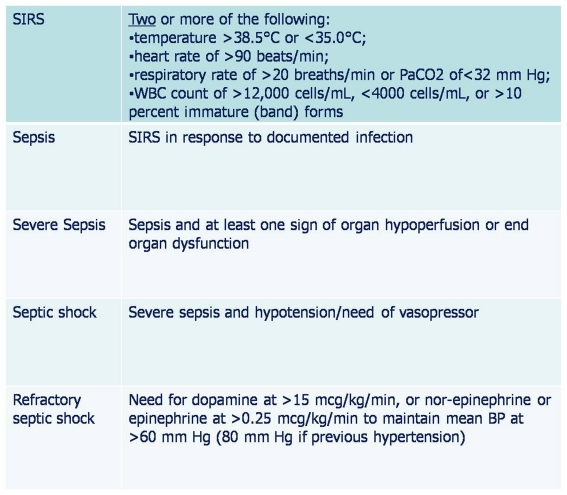
Definitions used to describe the condition of septic patients (Adapted from American college of chest physicians and society of critical care medicines surviving sepsis campaign published in 2008)

American college of chest physicians and society of critical care medicine have proposed definitions of sepsis and related syndromes (Fig. 3); and these are widely used while stratifying the risks for hospitalized patients [20]. These definitions of systemic inflammatory response syndrome (SIRS), sepsis, severe sepsis, and septic shock are based on clinical experience and the correlation of infection progression with appropriate physiologic responses.

EGDT has shown to increase survival by 20 % [10], and should be initiated as soon as the diagnosis is suspected. This include invasive hemodynamic monitoring with the aim of maintaining: central venous pressure (CVP) between 8 and 12 mm Hg, mean arterial pressure 65 mm Hg, urine output 0.5 mL/kg/hr, and central venous or mixed venous oxygen (SvO_2_) saturation 70%. If the SvO_2_ is less than 70% after maintain CVP of 8-12 mm Hg, infusion of packed red blood cells is indicated to maintain hematocrit of at least 30%. Dobutamine infusion is also indicated if cardiac output is compromised [10, 20]. Appropriate cultures should be obtained before starting antibiotics.

Intravenous antibiotics should be started as early as possible and always within the first hour of recognizing severe sepsis and septic shock [20]. The site of infection and responsible microorganisms are usually not known initially in a patient with sepsis. Antibiotic treatment must be guided by the patients susceptibility group and local knowledge of bacterial resistance [21]. Intravenous broad spectrum antibiotics directed against both gram positive and gram negative bacteria should be administered immediately after appropriate cultures have been obtained. Few guidelines exist for the initial selection of empiric antibiotics. If pseudomonas is an unlikely pathogen, combination of vancomycin with either the third or fourth generation cephalosporin, or beta-lactam/beta-lactamase inhibitor, or carbapenem is an excellent initial choice. If pseudomonas is a possible pathogen, combination of vancomycin and two antipseudomonal agents should be considered. Antifungal agent may be considered initially if risk factors for fungal infection are present, or within 48 hours if no improvement occurs [21]. Antimicrobial regimen should be reassessed in 48-72 hours on the basis of microbiological and clinical data [22]. Antibiotics should be stopped if the cause is found to be non-infectious.

Intravenous hydrocortisone is indicated for adult septic shock when hypotension responds poorly to adequate fluid resuscitation and vasopressors. Hydrocortisone dose should be ≤ 300 mg/day. Steroids are not recommended to treat sepsis in the absence of shock unless the patients endocrine or corticosteroid history warrants it [23]. Recombinant human activated protein C should be considered in adult patients with sepsis-induced organ dysfunction with clinical assessment of high risk of death and if there are no contraindications [24].

Our case emphasizes the importance of early recognition and management of septic shock. Gram negative septicemia is notorious for rapid deterioration due to endotoxin release. Multi-organ damage due to septic shock carries poor prognosis, and such patients should be managed aggressively with multidisciplinary approach. This case also highlights the fact that despite optimized treatment, this entity has very high mortality rates as shown in the previous studies. Nonetheless, early recognition, EGDT, and initiation of intravenous antibiotics are key components in treating patients with sepsis and septic shock.
